# Does Fever Caused by the COVID-19 Virus Before Labor Increase the Rate of MSAF and Affect Maternal and Fetal Outcomes?

**DOI:** 10.1155/2024/8456910

**Published:** 2024-11-11

**Authors:** Runrun Feng, Yu Tao, Haiyan Sun, Cen Cao, Hairong Gu, Junmei Hu, Wenwen Chang, Xia Li, Ziyan Jiang

**Affiliations:** ^1^Department of Obstetrics, The First Affiliated Hospital of Nanjing Medical University, Nanjing 210036, China; ^2^Department of Obstetrics, Taixing People's Hospital, Taizhou 225400, Jiangsu, China; ^3^Department of Obstetrics, Donghai People's Hospital, Lianyungang 222000, Jiangsu, China; ^4^Department of Obstetrics, Lishui People's Hospital, Nanjing 210036, Jiangsu, China; ^5^Department of Obstetrics, Maternal and Child Health Hospital of Huaian, Huaian 223001, Jiangsu, China

**Keywords:** COVID-19, fever, high-risk pregnancy, MSAF

## Abstract

**Objective:** The main objective of this study is to investigate whether there is a difference in the occurrence of MSAF between fever and nonfever–pregnant women during the COVID-19 pandemic.

**Study Design:** We conducted a multicenter retrospective study including pregnant women during the COVID-19 pandemic. Among the 400 pregnant women included in the final data analysis, 238 had fever during delivery, while 162 nonfever–pregnant women met the inclusion and exclusion criteria and served as controls. We collected various obstetric and neonatal parameters for both groups of patients, compared, and statistically tested the significance of these parameters. For parameters with significant statistical differences and clinical significance, we performed logistic regression analysis to explore potential risk factors for MSAF.

**Result:** In a sample of 400 parturients, a total of 69 individuals (17.25%) were discovered to have MSAF with the prevalence increasing to 21.85% in the fever population. A statistically significant association was observed between fever during pregnancy and MSAF, with a higher risk of development observed in fever–pregnant women compared to nonfever ones. Specifically, the odds of developing MSAF increased by a factor of 0.979 in fever-pregnant women compared to nonfever ones, as determined by a logistic regression model (OR = 1.979, 95% CI = 1.061∼3.693, *p*=0.032). Moreover, pregnant women with COVID-19 infection had a significantly higher risk of developing MSAF, with the odds increasing by a factor of 2.567 compared to uninfected pregnant women (OR = 3.567, 95% CI = 1.622∼7.845, *p*=0.002). In addition, the study also identified abnormal fetal heart monitoring (*p* < 0.05) and gestational age (*p* < 0.05) as independent risk factors for the occurrence of MSAF.

**Conclusion:** For pregnant women infected with COVID-19, the rate of MSAF disturbance significantly increases, therefore, it is necessary to pay more attention to fetal heart changes and amniotic fluid conditions, and actively managing labor is beneficial for improving delivery outcomes.

## 1. Introduction

Amniotic fluid (AF) is a liquid that surrounds the fetus within the amniotic sac, providing a low-resistance, thermally stable, and safe environment for fetal development. AF is composed of the fetus's surface on the amniotic membrane, the placenta, fetal skin, and fetal urine secretions. Meconium is a viscous, dark green substance that contains AF, fetal hair, bile, mucus, and epithelial cells. It is normally expelled by the newborn. When meconium is expelled by the fetus and enters the AF, it results in meconium-stained AF (MSAF). The precise pathophysiological mechanisms underlying the passage of meconium into the AF, as well as the resulting consequences of meconium aspiration, remain elusive. The passage of meconium is likely associated with the development of the fetal gastrointestinal system. An alternative theory suggests that the presence of meconium in utero may be associated with pathological mechanisms such as hypoxia-induced stress or infection. Reduced clearance of defecated meconium due to fetal swallowing impairment or unidentified placental dysfunction may also contribute to the presence of MSAF in cases of fetal hypoxia, in addition to or instead of an increase in meconium passage [1]. MSAF is usually found in the delivery room and birthing center and is associated with meconium aspiration syndrome [1–3], sepsis [4], and subsequently leads to neonatal cerebral palsy [5], amnionitis [6], chorioamnionitis, and postpartum endometritis [7]. MSAF is a manifestation of fetal distress in the uterus, which is associated with increased morbidity and mortality rates among neonates [8]. Intra-amniotic meconium staining is also associated with higher rates of instrument-assisted delivery, cesarean section, low birth weight, fetal distress, neonatal ICU admission, and neonatal mortality [9].

During the COVID-19 pandemic, we became concerned about the impact of fever on MSAF. Research has revealed that the occurrence rate of MSAF typically falls between 12% and 16% [10]. Our study found that the incidence of MSAF was 17.25%, with 69 pregnant women (or 17.25% of the sample) diagnosed as having this condition. The high frequency of MSAF in our study population may indicate an increase in the prevalence of fever patients due to viral infections during the COVID-19 pandemic, which could potentially exacerbate the incidence rates of this condition. Thus, our multicenter retrospective investigation was undertaken with the goal of identifying the risk factors associated with MSAF. The outcomes of our study are beneficial in enhancing the management of fever in pregnant women, improving the efficacy of intervention for critical illnesses related to MSAF, and considerably reducing healthcare expenditures.

## 2. Materials and Methods

### 2.1. Data Collection

The study on “Can fever lead to a higher risk of MSAF in COVID-19 infected women?” is a multicenter retrospective research. The research subjects (*n* = 636) were selected pregnant women who gave birth at the First Affiliated Hospital with Nanjing Medical University, Maternal and Child Health Hospital of Huai'an City, Jiangsu Province, Taixing People's Hospital, Donghai People's Hospital, and Lishui People's Hospital during the COVID-19 pandemic period (December 2022–January 2023). The result data were obtained through the clinical paper records of the participants and the hospital's electronic records, and necessary confirmations were made by the attending physician when needed. As this was a retrospective study, and the personal information and privacy of the enrolled pregnant women were anonymized during the analysis, there was no need for informed consent or inclusion of the population.

The inclusion criteria are as follows: (1) Age between 18 and 45 years-old. (2) Gestational age greater than 28 weeks at the time of delivery. (3) Pregnant women admitted during the COVID-19 pandemic (December 2022–January 2023).

Exclusion criteria included the following: (1) Stillbirth (*n* = 2). (2) Incomplete data on gestational age during delivery (*n* = 23). (3) Gestational age at delivery less than 28 weeks (*n* = 22). (4) Multiple pregnancies with twins or higher order multiples (*n* = 17).

This study divided 400 parturients into a study group (238 cases with the highest body temperature of ≥ 37.5°C) and a control group (162 cases with the highest body temperature of < 37.5°C) with a critical point of the highest body temperature of 37.5°C (axillary temperature). The two groups of pregnant women were compared for the situation of AF. We analyzed the maximum maternal temperature, fever time, COVID-19 nucleic acid presence, age, body mass index (BMI), gestational age, gravidity, parity, primipara, gestational diabetes mellitus (GDM), pregnancy-induced hypertension (PIH), preeclampsia, cord or placental abnormalities, premature rupture of membrane (PROM), with positive Group B *Streptococcus* (GBS), other diseases, placental abruption, spontaneous conception, *in vitro* fertilization and embryo transfer (IVF-ET), intrauterine insemination (IUI), maternal outcomes, MSAF, AF volume, artificial rupture of membrane, episiotomy, with intrapartum regional analgesia, with abnormal FHR, mode of delivery, 2-h vaginal bleeding volume, 24-h vaginal bleeding volume, the latest routine blood test before delivery, and neonatal outcomes of each group of pregnant women.

The main outcome of this study is MSAF, which is defined as when the fetus excretes meconium into the AF due to various intricate factors. The baseline characteristics of population statistics include the maximum maternal temperature (°C), fever time (h), COVID-19 nucleic acid presence, age (year), BMI (kg/m^2^), gestational age (days), gravidity, parity, and primipara. Medical and obstetric factors include the following: pregnancy complications, assisted reproductive technology (ART), maternal outcomes, the latest routine blood test before delivery, and neonatal outcomes. There are three ARTs as follows: spontaneous conception, IVF-ET, and IUI. Among these, pregnancy complications include PIH, GDM, preeclampsia, cord or placental abnormalities, and PROM, with positive GBS, placental abruption, and other diseases. PIH is defined as systolic blood pressure (SBP) of > 140 mmHg and diastolic blood pressure (DBP) of > 90 mmHg [11]. GDM is defined as a glucose tolerance disorder with onset during pregnancy [12]. The diagnostic criteria for preeclampsia are newly developed hypertension (SBP ≥ 140 mmHg and/or DBP ≥ 90 mmHg) and proteinuria (> 300 mg/24 h) after 20 weeks of gestation in women with previously normal blood pressure [13]. PROM is defined as the rupture of the membranes before the onset of labor [14]. Placental abruption refers to the abnormal separation after 20 weeks of gestation and prior to birth. Abnormal FHR refers to the situation where the fetal heart rate monitor shows fetal heart rate deceleration during the antenatal period.

### 2.2. Data Processing

This study employed SPSS 26.0 software for statistical analysis. The normality of sample data is determined by using the Shapiro normality test for continuous (quantitative) data. If the data conform to a normal distribution, the mean ± standard deviation (SD) is used to represent it. Independent sample *t*-tests are used for comparing the two groups. If the data do not follow a normal distribution, the median (25th percentile and 75th percentile) will be used to represent the data. The comparison between the two groups will be analyzed using the Wilcoxon test. Categorical data are statistically described using frequency (percentage), and intergroup comparisons are conducted using the *χ*2 test or Fisher's exact test. When the two-sided *p* value is less than 0.05, the difference is considered to be statistically significant.

### 2.3. Statistical Analysis

In this study, we initially conducted a univariate analysis in order to group the data according to variables such as maternal fever or COVID-19 infection. Those variables that displayed both statistical significance and clinical relevance were included in the binary logistic regression analysis. This specific analysis was then carried out separately for both maternal fever and maternal COVID-19 infection outcomes. The purpose of this analysis was to determine the primary cause of fever among the population of this study with COVID-19 infection.

**Figure 1:**
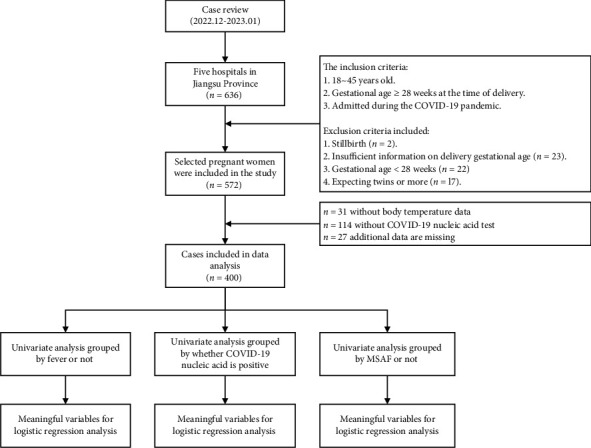
The flow of the present study design.

Subsequently, we performed another univariate analysis by grouping the data according to the presence or absence of AF or meconium staining. Similar to the previous analysis, any variables that exhibited statistical differences and clinical significance were further examined using logistic regression analysis. The goal of this analysis was to investigate the relationship between maternal fever and the presence of MSAF (Figure 1).

## 3. Result

The distribution of patients is shown in Table 1. Among the 400 pregnant women, 69 (17.25%) were diagnosed with MSAF. Among the fever group (238 cases), 52 (21.85%) pregnant women had MSAF, while among the nonfever group (162 cases), 17 (10.49%) pregnant women had MSAF. There was a statistically significant difference in MSAF between the two groups (*p* < 0.05). A detailed analysis was conducted on the distinctive clinical data features of both groups. It was found that factors such as maximum maternal temperature (*p* < 0.001), fever time (*p* < 0.001), COVID-19 nucleic acid presence (*p* < 0.001), BMI (*p* < 0.036), gestational age (*p*=0.001), gravidity (*p* < 0.001), parity (*p* < 0.001), primipara (*p* < 0.001), PIH (*p*=0.004), GDM (*p*=0.04), PROM (*p* < 0.001), with positive GBS (*p* < 0.001), 2-h vaginal bleeding volume (*p* < 0.001), 24-h vaginal bleeding volume (*p*=0.019), artificial rupture of membrane (*p* < 0.001), episiotomy (*p* < 0.049), with intrapartum regional analgesia (*p* = 0.049), with abnormal FHR (*p* < 0.001), neutrophil count (*p* < 0.001), neutrophil percentage (%) (*p* < 0.001), and an Apgar score of 1 min (*p* < 0.001) also exhibited differences.

Table 2 presents the results of logistic regression analysis carried out to investigate potential factors responsible for inducing fever. The clinical practice observed during the study period confirmed that COVID-19 positivity (*p* < 0.001) was identified as a significant fever-causing factor. In addition, the analysis highlighted that intrapartum regional analgesia (*p* < 0.05) was a pertinent risk factor for fever.

Table 3 presents the detailed clinical characteristics and the results of univariate analysis between two groups classified by the COVID-19 nucleic acid test results. Risk factors associated with COVID-19 nucleic acid positivity were found in the table, including fever, maximum maternal temperature, fever time, gravidity, parity, primipara, 2-h vaginal bleeding volume, MSAF, *N* count, *N* (%), with positive GBS, and an Apgar score of 1 min.

The findings of our logistic regression analysis on potential predictors of COVID-19 positivity are presented in Table 4. Our results indicate that fever demonstrated a statistically significant association with COVID-19 positivity, while none of the other variables exhibited a significant correlation. Taken together, our findings suggest that the presence of fever may be the sole indicator of COVID-19 positivity among our study participants.

Table 5 presents a comprehensive summary of the clinical characteristics and findings obtained through univariate analysis on two distinct patient groups, stratified based on the presence or absence of MSAF. The results of this analysis indicate that factors such as fever, COVID-19 nucleic acid presence, gestational age, gravidity, parity, primipara, other diseases, artificial rupture of membrane, 24-h vaginal bleeding volume, with abnormal FHR, *N* count, *N* (%), and with positive GBS are significantly associated with the occurrence of MSAF.

After conducting logistic regression analysis to account for the various factors in Table 6, it was determined that fever–pregnant women had a higher risk of MSAF compared to nonfever–pregnant women at a ratio of 1.979 (*p*=0.032). Further analysis indicated a correlation between MSAF and abnormal fetal heart rate monitoring (*p*=0.007), with the risk of meconium staining increasing as gestational age increased (*p*=0.005, OR = 1.049).

Table 7 performed a multivariable logistic regression analysis to control for the positivity of nucleic acid detection. The results of the analysis indicated that the likelihood of MSAF in COVID-19 positive patients was 2.567 times greater than in negative patients (*p*=0.002). In addition, the relative risks of abnormal fetal heart monitoring and gestational age were 2.568 (*p*=0.003) and 1.055 (*p*=0.002), respectively.

## 4. Discussion

This study analyzes and compares the occurrence of MSAF and differences in maternal and neonatal outcomes between fever–pregnant and nonfever–pregnant women due to COVID-19. Results reveal a significant increase in the incidence of MSAF in fever–pregnant women compared to nonfever–pregnant women. During the COVID-19 pandemic, the incidence of fever in pregnant women has significantly increased. Consistent with previous findings, this study observed a significant increase in the likelihood of fever among individuals infected with COVID-19 compared to those who were uninfected [15–21].

Nonetheless, concurrently in this research, we discovered that intrapartum regional analgesia acts as an independent risk factor for developing maternal fever. Analogous observations were posited in Segal's scholarly study, in which there exists a marked correlation between epidural analgesia administered during parturition and maternal fever [22]. However, another study also demonstrated that only about 15%–25% of patients receiving epidural anesthesia during delivery would experience relevant maternal fever symptoms [23]. Returning to our study, while regional anesthesia during delivery is an independent risk factor for maternal fever, in our study cohort, on one hand, the incidence of maternal fever in those who received labor analgesia increased significantly to as high as 57%. On the other hand, fever attributed to labor analgesia usually occurs during the delivery process, while in our study, the majority of pregnant women had a fever before delivery, with only a few experiencing it during delivery. As a result, we believe that fever induced by labor analgesia in this study did not have a significant impact on the outcomes. Furthermore, the relationships between COVID-19 and other pregnancy complications were also analyzed, and it was found that only MSAF and maternal fever showed significant differences. Therefore, in this study, COVID-19 infection was closely related to MSAF and maternal fever.

There is a close relationship between maternal fever and the increase in the incidence of MSAF. The findings of this investigation have highlighted such a connection. The high incidence of MSAF in COVID-19 positive patients is not coincidental. A retrospective cohort study from Taiwan showed an incidence rate of 22% for MSAF among their study population of COVID-19 positive pregnant women [24]. Similarly, similar numbers were also reported in the study conducted by Nayak et al. [25]. Furthermore, according to a research conducted at the University of Vigo in Europe, the incidence of fever during childbirth is higher in populations with MSAF [26], Concurrently, maternal fever during labor poses a crucial risk factor for MSAF.

Besides fever, fetal heart pattern and gestational age were also noted to be independent risk factors of MSAF. Through our investigation, we have also found significant differences in the occurrence of abnormal fetal heart monitoring among pregnant women with MSAF compared to those with clear AF. We observed that an abnormal fetal heart monitoring during labor (Category II monitoring) yielded a significantly increased risk of 1.382 times compared to pregnant women with Category I fetal heart monitoring. The observation that the frequency of anomalous fetal cardiac monitoring elevates progressively with the advancement of MSAF is also discernible in Rodriguez Fernandez et al.'s study [26]. Likewise, Adnan et al. [27] have reported comparable findings. Their study reveals that expectant mothers with MSAF exhibit a higher frequency of abnormal fetal heart monitoring, suggesting a significant association between fetal heart irregularities and AF discoloration caused by meconium. This reminds us that abnormal fetal heart monitoring may be used as an indicator for the occurrence of MSAF. The increase in the incidence of MSAF may also be associated with gestational age. In this study, pregnant women who had MSAF generally had a longer gestational age. A study conducted at a Nigerian University Teaching Hospital also found that as the gestational age increases, the incidence of MSAF also increases [28].

It should be noted that the inclusion of additional diseases has a highly significant impact on the risk of fecal contamination. In this investigation, we have categorized a range of other pregnancy complications, including hyperthyroidism, hypothyroidism, anemia, and intrahepatic cholestasis of pregnancy, as pregnancy-associated other diseases, while excluding preeclampsia, GDM, and PIH. Due to constraints on the size of our clinical sample, we were unable to carry out specific classification and analysis of these factors. This is largely due to the scarcity of available clinical data, which could introduce substantial biases. Nonetheless, literature sources have indicated that these factors remain strongly associated with fecal contamination of the AF. For instance, the research conducted by L. Monen indicates that among women delivering at a gestation period of 41 weeks or more, there exists an independent association between elevated TSH levels and the presence of MSAF [29]. Consequently, future research will continue to examine these factors in greater detail.

To sum up, our study found that maternal fever caused by COVID-19 is an important risk factor for MSAF. In addition, abnormalities in fetal heart monitoring and prolonged gestational age are also associated with increased incidence of MSAF. Nonetheless, there is no influence on the maternal pregnancy outcomes, including the frequency of assisted delivery, caesarean section, and postpartum hemorrhage. In addition, there are no repercussions on newborn Apgar scores with regard to pediatric outcomes. It is therefore plausible to presume that even if maternal fever due to COVID-19 results in MSAF, it does not impinge on the maternal and neonatal pregnancy outcomes. Consequently, it does not affect the decision-making process of the clinicians. The recommended approach in clinical settings is symptomatic treatment, including alleviating fever, providing oxygen therapy, and fluid replacement. Nonetheless, pregnant women who have MSAF remain categorized as a high-risk group. Thus, it is recommended that pregnant women with risk factors for meconium staining, such as maternal fever as described in this investigation, undergo delivery attended by close fetal surveillance by a certified team with resuscitation skills [30].

## Figures and Tables

**Table 1:** The differences between pregnant women with and without fever.

	Non-fever (*n* = 162)	Fever (*n* = 238)	*P* value
Baseline characteristics			
Maximum maternal temperature (°C), median (IQR)	**36.8 (36.7, 37)**	**38.2 (37.8, 38.6)**	**< 0.001**
Fever time (h), median (IQR)	**0 (0, 0)**	**16 (8, 30.5)**	**< 0.001**
COVID-19 nucleic acid, n (%)	**80 (49.38%)**	**204 (85.71%)**	**< 0.001**
Age (year), median (IQR)	**30 (26, 33)**	**29 (27, 32)**	**0.688**
BMI (kg/m^2^), median (IQR)	**27.99 (25.95, 31.25)**	**27.34 (25.24, 29.72)**	0.036
Gestational age (days), median (IQR)	**273.5 (268, 279)**	**277 (271, 282)**	**0.001**
Gravidity, median (IQR)	**2 (1, 3)**	**1 (1, 2)**	**< 0.001**
Parity, median (IQR)	**2 (1, 2)**	**1 (0, 1)**	**< 0.001**
Primipara, *n* (%)	**104 (64.2%)**	**47 (19.75%)**	**< 0.001**
Complications, *n* (%)			
GDM	**19 (11.73%)**	**46 (19.49%)**	**0.04**
Preeclampsia	**4 (2.47%)**	**9 (3.8%)**	**0.463**
Cord or placental abnormalities	**6 (3.73%)**	**6 (2.59%)**	**0.728**
PROM	**3 (1.85%)**	**28 (11.86%)**	**< 0.001**
With positive GBS	**4 (2.47%)**	**27 (11.34%)**	**< 0.001**
PIH	**2 (1.24%)**	**18 (7.59%)**	**0.004**
Other diseases	**11 (6.79%)**	**27 (11.34%)**	**0.127**
Placental abruption	**5 (3.09%)**	**6 (2.53%)**	**0.983**
ART, *n* (%)			
Spontaneous conception	**149 (96.13%)**	**211 (92.54%)**	**0.372**
IVF-ET	**6 (3.87%)**	**15 (6.58%)**	
IUI	**0 (0%)**	**2 (0.88%)**	
Maternal outcomes			
MSAF, *n* (%)	**17 (10.49%)**	**52 (21.85%)**	**0.003**
AF volume (mL), median (IQR)	**500 (400, 500)**	**500 (400, 500)**	**0.102**
Artificial rupture of membrane, *n* (%)	**119 (73.46%)**	**133 (56.12%)**	**< 0.001**
Episiotomy, *n* (%)	**10 (6.21%)**	**29 (12.18%)**	**0.049**
With intrapartum regional analgesia, *n* (%)	**20 (36.36%)**	**136 (57.38%)**	**0.005**
With abnormal FHR, *n* (%)	**11 (6.79%)**	**60 (25.32%)**	**< 0.001**
Mode of delivery, *n* (%)			**0.195**
Vaginal	**91 (56.17%)**	**118 (49.58%)**	
	**71 (43.83%)**	**120 (50.42%)**	
2-h Vaginal bleeding volume (mL), median (IQR)	**286.30 ± 117.32**	**325.82 ± 128.78**	**0.002**
24-h Vaginal bleeding volume (mL), median(IQR)	**197.36 ± 196.83**	**304.44 ± 257.42**	**0.018**
The latest routine blood test before delivery, median (IQR)			
WBC count (10^9^/L)	**7.94 (6.76, 9.46)**	**8.31 (7.04, 10.37)**	**0.101**
CRP (mg/L)	**1.08 (0, 6.02)**	**1.49 (0, 5.7)**	**0.696**
N (%)	**73 (69.45, 77.93)**	**75.15 (70.57, 83.12)**	**0.002**
N count (10^9^/L)	**5.88 (4.87, 7.15)**	**6.34 (5.1, 8.12)**	**0.012**
Neonatal outcomes			
Male/female, *n* (%)	**77 (47.53%)/85 (52.47%)**	**124 (52.1%)/111 (46.64%)**	**0.211**
Birth weight (g), median (IQR)	**3395 (3100, 3650)**	**3450 (3150, 3700)**	**0.227**
Apgar score of 1 min	**10 (10, 10)**	**10 (9, 10)**	**< 0.001**
Apgar score of 5 min	**10 (10, 10)**	**10 (10, 10)**	**1**

Abbreviation: N, neutrophil.

**Table 2:** Testing positive for COVID-19 nucleic acid and intrapartum regional analgesia are independent risk factors for maternal fever.

	OR	95% CI for OR		*P* value
COVID-19 nucleic acid	3.883	1.824	8.262	< 0.001
Gestational age	1.012	0.984	1.04	0.401
BMI	1.021	0.926	1.125	0.683
PIH	0.038	0.001	1.409	0.076
DM	147975924	0	.	0.999
PROM	63401904	0	.	0.999
With intrapartum regional analgesia	83.775	10.804	649.585	< 0.001
With positive GBS	1.74	0.481	6.29	0.398

**Table 3:** The differences between pregnant women who tested positive for COVID-19 and those who tested negative for COVID-19.

	COVID-19 nucleic acid negative (*n* = 116)	COVID-19 nucleic acid positive (*n* = 284)	*P* value
Baseline characteristics			
Maternal fever, *n* (%)	**34 (29.31%)**	**204 (71.83%)**	**< 0.001**
Maximum maternal temperature (°C), median (IQR)	**37 (36.7, 37.8)**	**38 (37.2, 38.5)**	**< 0.001**
Fever time (h), median (IQR)	**0 (0, 4)**	**11 (0, 24)**	**< 0.001**
Age (year), median (IQR)	**30 (26, 33)**	**29 (26, 32)**	**0.688**
Gestational age (days), median (IQR)	**276 (269.75, 281)**	**276 (269, 281)**	**0.804**
Gravidity, median (IQR)	**2 (1, 3.25)**	**2 (1, 2)**	**0.001**
BMI (kg/m^2^), median (IQR)	**27.99 (25.56, 30.45)**	**27.39 (25.47, 29.86)**	**0.422**
Parity, median (IQR)	**2 (1, 2)**	**1 (1, 1.25)**	**< 0.001**
Primipara, *n* (%)	**59 (50.86%)**	**92 (32.39%)**	**0.001**
Complications, *n* (%)			
DM	**15 (12.93%)**	**50 (17.73%)**	**0.239**
Preeclampsia	**4 (3.48%)**	**9 (3.17%)**	**1**
Cord or placental abnormalities	**2 (1.75%)**	**10 (3.58%)**	**0.526**
PROM	**8 (6.96%)**	**23 (8.13%)**	**0.693**
With positive GBS	**7 (6.03%)**	**24 (8.45%)**	**< 0.001**
PIH	**2 (1.72%)**	**18 (6.38%)**	**0.053**
Other diseases	**4 (3.45%)**	**34 (11.97%)**	**0.008**
Placental abruption	**3 (2.59%)**	**8 (2.83%)**	**1**
ART, *n* (%)			
Spontaneous conception	110 (94.83%)	250 (93.63%)	0.073
IVF-ET	4 (3.45%)	17 (6.37%)	
IUI	2 (1.72%)	0 (0%)	
Maternal outcomes			
MSAF, *n* (%)	8 (6.9%)	61 (21.48%)	< 0.001
AF volume (mL), median (IQR)	500 (400, 500)	500 (400, 500)	0.348
Artificial rupture of membrane, *n* (%)	78 (67.83%)	174 (61.27%)	0.219
Episiotomy, *n* (%)	9 (7.83%)	30 (10.56%)	0.404
With intrapartum regional analgesia, *n* (%)	34 (64.15%)	122 (51.05%)	0.084
With abnormal FHR, *n* (%)	14 (12.07%)	57 (20.14%)	0.056
Mode of delivery, *n* (%)			0.064
Vaginal	69 (59.48%)	140 (49.3%)	
Cesarean	47 (40.52%)	144 (50.7%)	
2-h Vaginal bleeding volume (mL), median(IQR)	285.57 ± 121.14	319.71 ± 126.28	0.013
24-h Vaginal bleeding volume (mL), median(IQR)	234.03 ± 211.53	298.83 ± 257.56	0.152
The latest routine blood test before delivery, median (IQR)			
WBC count (10^9^/L)	8.49 (7.26, 10.3)	8.05 (6.67, 9.88)	0.073
CRP (mg/L)	0.57 (0, 2.74)	1.57 (0, 6.02)	0.177
N (%)	73.95 (70.07, 78.35)	74.85 (69.7, 82.3)	0.210
N count (10^9^/L)	6.28 (5.45, 8.13)	6.12 (4.83, 7.74)	0.123
Neonatal outcomes			
Male/female, *n* (%)	59 (50.86%)/57 (49.14%)	142 (50%)/139 (48.94%)	0.783
Birth weight (g), median (IQR)	3420 (3050, 3692.5)	3435 (3150, 3650)	0.815
Apgar score of 1 min	10 (10, 10)	10 (10, 10)	0.001
Apgar score of 5 min	10 (10, 10)	10 (10, 10)	1

Abbreviation: N, neutrophil.

**Table 4:** The only independent factor associated with testing positive for COVID-19 is maternal fever.

	OR	95% CI for OR		*P* value
Maternal fever	4.691	2.661	8.27	< 0.001
Other diseases	0.464	0.147	1.47	0.192
With positive GBS	0.707	0.275	1.816	0.472
MSAF	2.188	0.937	5.105	0.07

**Table 5:** Differences between meconium-stained amniotic fluid and clear amniotic fluid in pregnant women.

	Clear AF (*n* = 331)	MSAF (*n* = 69)	*P* value
Baseline characteristics			
Maternal fever, *n* (%)	186 (56.19%)	52 (75.36%)	0.003
Maximum maternal temperature (°C), median (IQR)	37.8 (36.8, 38.35)	37.8 (37.5, 38.3)	0.102
Fever time (h), median (IQR)	4 (0, 18)	8 (2, 16)	0.127
COVID-19 nucleic acid, *n* (%)	223 (67.37%)	61 (88.41%)	< 0.001
Age(year), median (IQR)	29 (26, 33)	29 (26.75, 30.25)	0.326
Gestational age (days), median (IQR)	275 (269, 280)	280 (275, 285)	< 0.001
Gravidity, median (IQR)	2 (1, 3)	1 (1, 2)	< 0.001
BMI (kg/m^2^), median (IQR)	27.59 (25.47, 30)	27.48 (25.48, 29.76)	0.684
Parity, median (IQR)	1 (1, 2)	0 (0, 1)	< 0.001
Primipara, *n* (%)	140 (42.3%)	11 (15.94%)	< 0.001
Complications, *n* (%)			
DM	58 (17.63%)	7 (10.14%)	0.126
Preeclampsia	11 (3.33%)	2 (2.9%)	1
Cord or placental abnormalities	10 (3.08%)	2 (2.94%)	1
PROM	29 (8.81%)	2 (2.9%)	0.095
With positive GBS	23 (6.95%)	8 (11.59%)	< 0.001
PIH	13 (3.94%)	7 (10.29%)	0.06
Other diseases	16 (4.83%)	22 (31.88%)	< 0.001
Placental abruption	10 (3.03%)	1 (1.45%)	0.745
ART, *n* (%)			
Spontaneous conception	297 (94.29%)	63 (92.65%)	0.699
IVF-ET	16 (5.08%)	5 (7.35%)	
IUI	2 (0.63%)	0 (0%)	
Maternal outcomes			
AF volume (mL), median (IQR)	500 (400, 500)	500 (400, 500)	0.619
Artificial rupture of membrane, *n* (%)	219 (66.36%)	33 (47.83%)	0.004
Episiotomy, *n* (%)	29 (8.79%)	10 (14.49%)	0.147
With intrapartum regional analgesia, *n* (%)	115 (51.57%)	41 (59.42%)	0.253
With abnormal FHR, *n* (%)	48 (14.55%)	23 (33.33%)	< 0.001
Mode of delivery, *n* (%)			0.419
Vaginal	176 (53.17%)	33 (47.83%)	
Cesarean	155 (46.83%)	36 (52.17%)	
2-h Vaginal bleeding volume (mL), median(IQR)	314.60 ± 131.04	286.81 ± 92.84	0.095
24-h Vaginal bleeding volume (mL), median(IQR)	320.09 ± 259.60	191.59 ± 199.64	< 0.001
The latest routine blood test before delivery, median (IQR)			
WBC count (10^9^/L)	8.2 (6.89, 9.96)	8.42 (6.82, 10.07)	0.866
CRP (mg/L)	1.5 (0, 5.69)	1.25 (0, 6.54)	0.75
N (%)	74.8 (69.75, 81.1)	75.1 (71, 80.1)	0.571
N count (10^9^/L)	6.19 (4.9, 7.9)	6.3 (5, 7.88)	0.784
Neonatal outcomes			
Male/female, *n* (%)	171 (51.66%)/158 (47.73%)	30 (43.48%)/38 (55.07%)	0.262
Birth weight (g), median (IQR)	3430 (3100, 3680)	3450 (3250, 3650)	0.594
Apgar score of 1 min	10 (10, 10)	10 (10, 10)	0.191
Apgar score of 5 min	10 (10, 10)	10 (10, 10)	1

Abbreviation: N, neutrophil.

**Table 6:** Maternal fever, with abnormal FHR and gestational age are independent risk factors for MSAF.

	OR	95% CI for OR		*P*value
Maternal fever	1.979	1.061	3.693	0.032
With abnormal FHR	2.382	1.268	4.473	0.007
Gestational age	1.049	1.014	1.085	0.005
PROM	0	0	.	0.999

**Table 7:** COVID-19 nucleic acid, with abnormal FHR and gestational age are independent risk factors for MSAF.

	OR	95% CI for OR		*P* value
COVID-19 nucleic acid	3.567	1.622	7.845	0.002
With abnormal FHR	2.568	1.372	4.808	0.003
Gestational age	1.055	1.02	1.092	0.002
PROM	0	0	.	0.999

## Data Availability

Research data are not shared.
